# P-1421. Impact of a Community Health Worker and Pharmacist Led Vaccine Gap Closure Program in Missouri

**DOI:** 10.1093/ofid/ofaf695.1608

**Published:** 2026-01-11

**Authors:** Adriana Gardner, John Galdo, Jasmine Gonzalvo, Annie Eisenbeis, Rakhi Karwa

**Affiliations:** Purdue University, Los Altos, California; Seguridad, Inc, Nashville, Tennessee; Purdue University, Los Altos, California; Missouri Pharmacy Association, Jefferson City, Missouri; Purdue University College of Pharmacy, Indianapolis, Indiana

## Abstract

**Background:**

Significant gaps in vaccination rates exist, with only 22.8% of US adults receiving all age-appropriate vaccines in 2022. As trusted community members, community health workers (CHWs) are uniquely positioned to address vaccine hesitancy and provide culturally appropriate services. A statewide Missouri community vaccination program led by pharmacists and CHWs was implemented to overcome barriers to vaccination by delivering vaccines outside clinic settings, addressing vaccine hesitancy, and covering costs when needed. This study analyzed the impact of this vaccination initiative.

**Figure:**
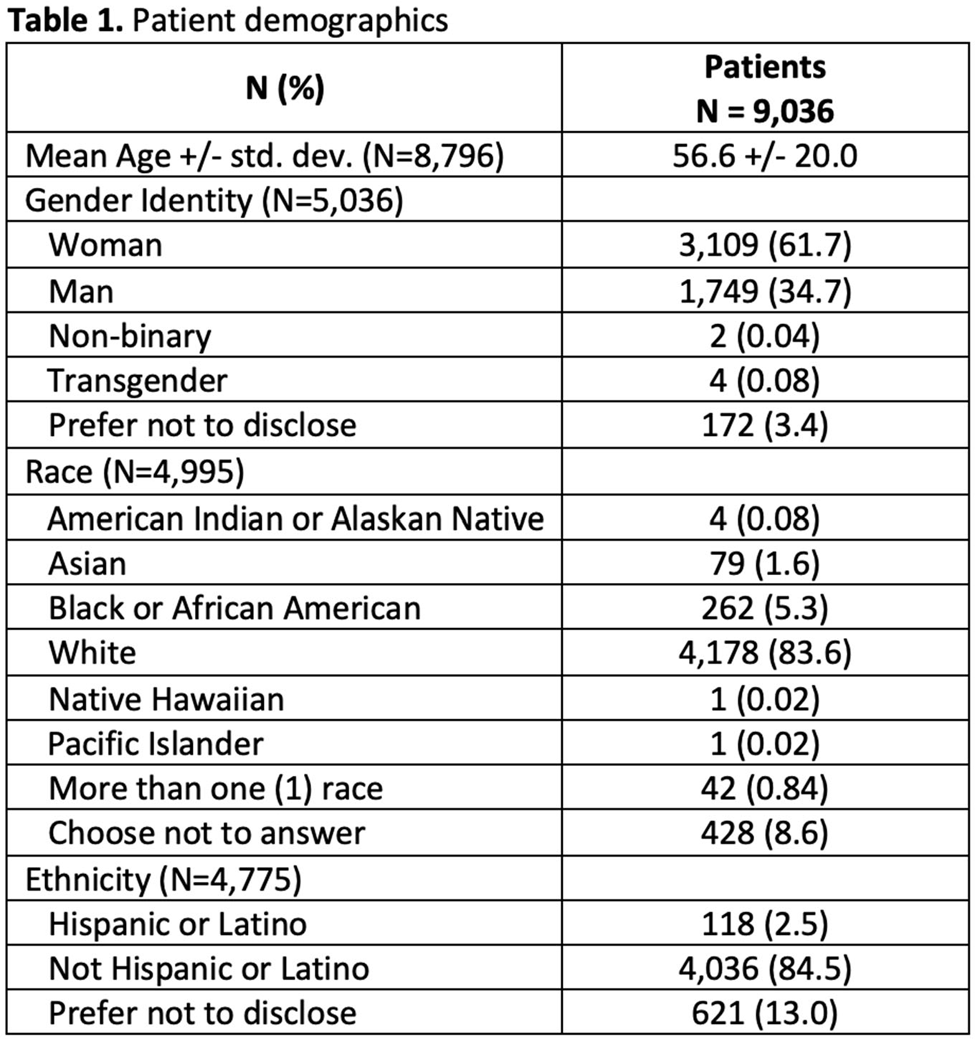

**Figure:**
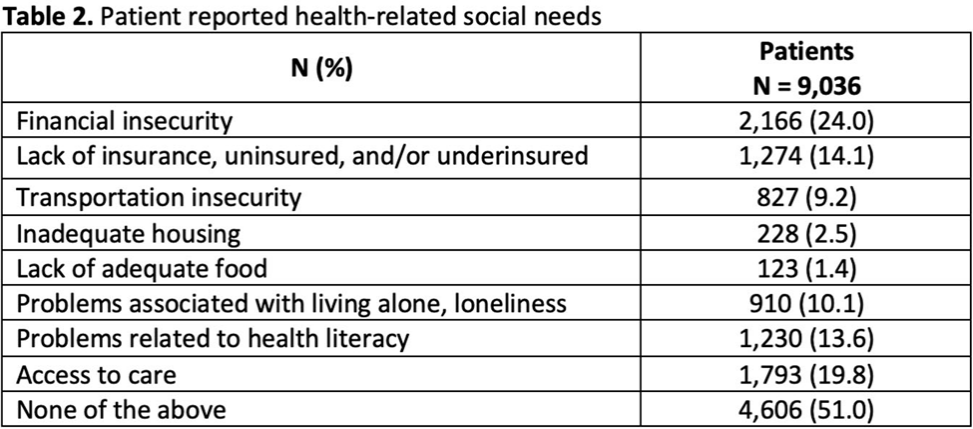

**Methods:**

A retrospective review of clinical records from the Missouri community vaccination program was conducted. Patients were included if they had ≥ 1 encounter with the program. Descriptive statistics were used to characterize patient demographics, social needs, and the types of vaccines given. The primary outcome was the percent of patients that received ≥ 1 vaccine. The secondary outcomes were to report the rate of vaccination among patients who received vaccine hesitancy counseling and to describe the insurance type associated with a cost barrier due to copays.

**Figure:**
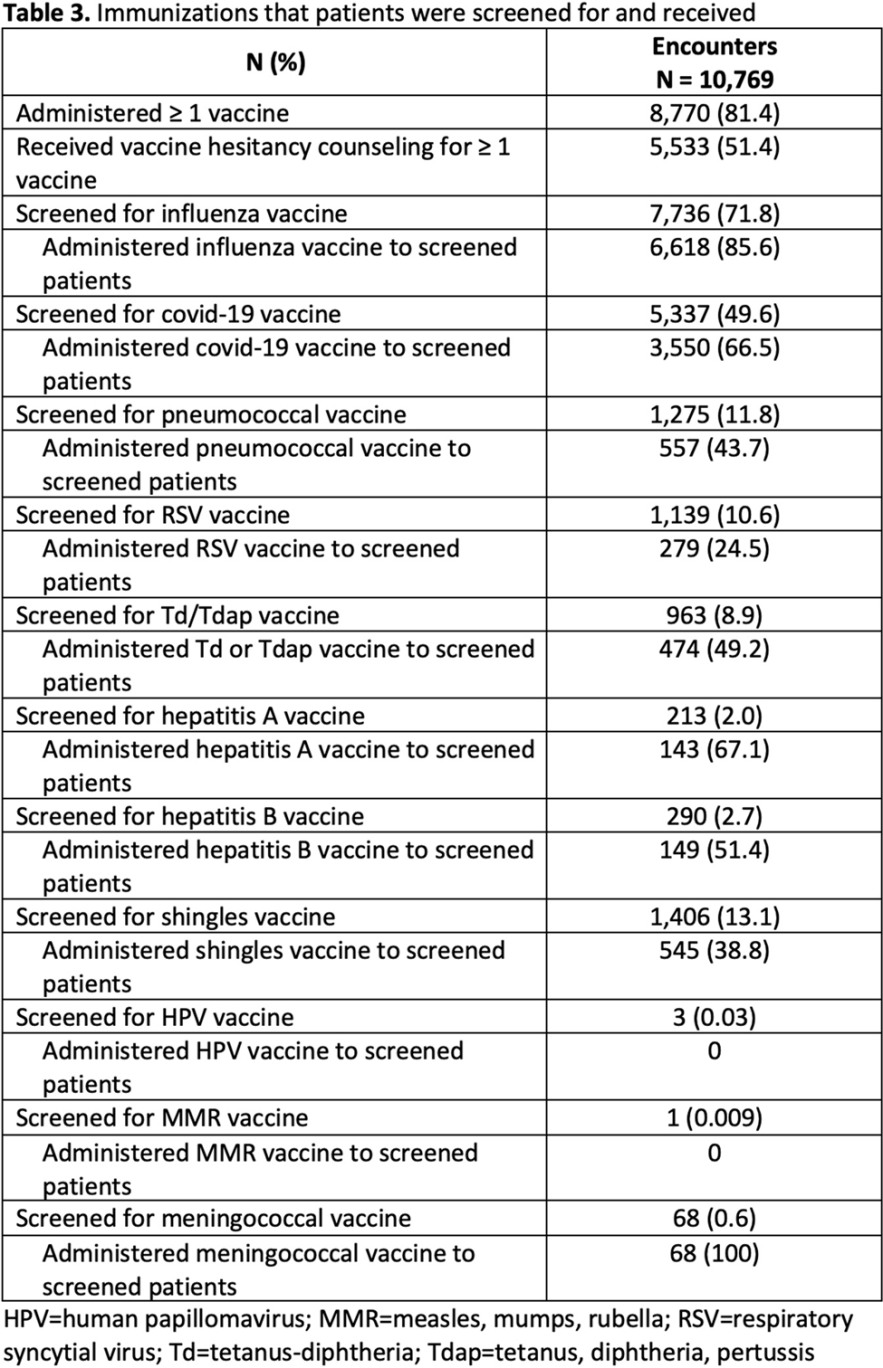

**Figure:**
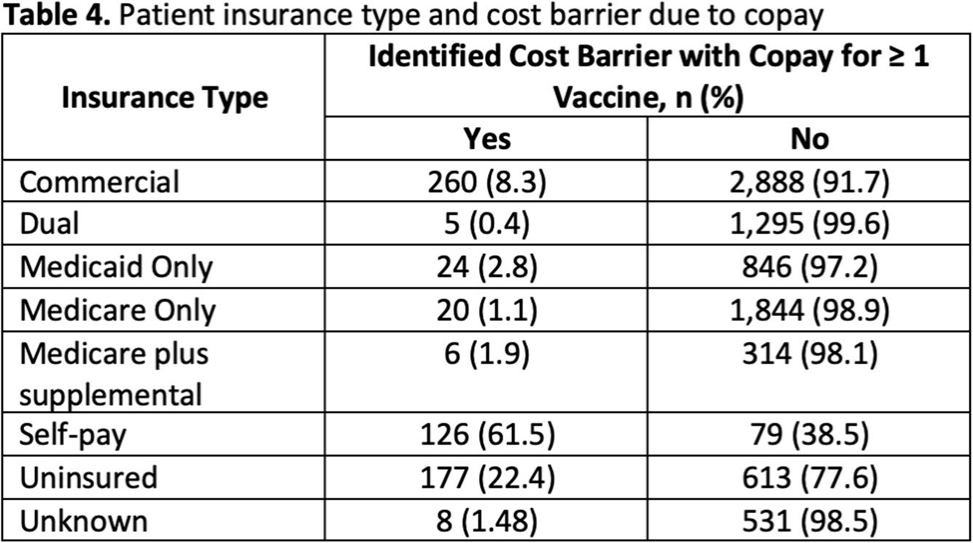

**Results:**

The vaccination program was held from September 2024 to October 2024 and included 9,036 patients with a mean age of 56.6 years (Table 1). Self-identified health-related social needs were most commonly financial insecurity (24%), limited access to care (20%), and lack of health insurance (14%) (Table 2). Patients received ≥ 1 vaccine and vaccine hesitancy counseling at 81.4% and 51.4% of encounters respectively (Table 3). In total, at the 10,769 encounters, 12,383 vaccines were administered. After vaccine hesitancy counseling, which was provided for 9,395 vaccines, vaccines were administered 64% of the time. The most frequently administered vaccines were influenza (n=6,618), covid-19 (n=3,550), pneumococcal (n=557), and shingles (n=545) vaccines. Cost barriers due to vaccination copays were most often reported among patients who are self-pay, uninsured, or have commercial insurance (Table 4).

**Conclusion:**

The Missouri community vaccination program led by pharmacists and CHWs was an effective program to help close vaccination gaps regardless of geography and socioeconomic status.

**Disclosures:**

John Galdo, PharmD, MBA, BCPS, BCGP, Eli Lilly: Employment, Spouse|Pfizer: Advisor/Consultant|Takeda: Honoraria Jasmine Gonzalvo, PharmD, Lilly: Advisor/Consultant|Lilly: Honoraria

